# Draft Genome Sequence of a Sequence Type 11 *Klebsiella pneumoniae* Clinical Strain Carrying a *bla*_KPC-2_ Carbapenemase Gene and an *rmtB* 16S rRNA Methylase Gene

**DOI:** 10.1128/genomeA.01549-16

**Published:** 2017-02-09

**Authors:** Zhihong Yao, Yu Feng, Li Wei, Zhiyong Zong

**Affiliations:** a Center of Infectious Diseases, West China Hospital, Sichuan University, Chengdu, China; b Division of Infectious Diseases, State Key Laboratory of Biotherapy, Chengdu, China; c Department of Infection Control, West China Hospital, Sichuan University, Chengdu, China; d Faculty of Pharmacology and Medical Technology, Hanzhong Vocational and Technical College, Hanzhong, Shaanxi, China

## Abstract

*Klebsiella pneumoniae* strain WCHKP649, recovered from a human wound, carried the carbapenemase gene *bla*_KPC-2_ and 16S rRNA methylase gene *rmtB*. Here, we report its 5.6-Mb draft genome sequence, comprising 171 contigs with an average 57.34% G+C content. The genome contained 5,284 coding sequences and 84 tRNA genes.

## GENOME ANNOUNCEMENT

*Klebsiella pneumoniae* strain WCHKP649 was recovered from the wound secretion of a car accident-injured patient in West China Hospital, Chengdu, China, in December 2015. Strain WCHKP649 exhibited high-level resistance to meropenem (MIC, 256 μg/ml) and amikacin (MIC, >512 μg/ml). Screening for acquired carbapenemase genes *bla*_GES_, *bla*_IMP_, *bla*_IMI_, *bla*_KPC_, *bla*_NDM_, *bla*_OXA-48_-like, and *bla*_VIM_ using PCR (1–4) found that strain WCHKP649 carried *bla*_KPC_. 

Genomic DNA of strain WCHKP649 was prepared using the QIAamp DNA minikit (Qiagen, Hilden, Germany) and then was sequenced using the HiSeq X10 sequencer (Illumina, San Diego, CA) with the 150-bp paired-end protocol and 200× coverage. A total of 5,620,928 reads and 1.69 Gb clean bases were generated. SPAdes (version 3.9) (5) was used for *de novo* assembly and generated 171 contigs with 103 contigs ≥1,000 bp in length (*N*_50_, 176,178 bp) and 57.34% G+C content. The genome size was about 5.6 Mb. Annotation of the genomic sequence was carried out using Prokka (version 1.11) (6). The genome of strain WCHKP649 contained 5,280 coding sequences and 84 tRNA genes. The sequence type (ST) of strain WCHKP649 was determined using the genome sequence to query the *K. pneumoniae* multilocus sequence typing (MLST) database (http://bigsdb.pasteur.fr/klebsiella/klebsiella.html). Strain WCHKP649 belonged to ST11, which has been found in Asia, Europe, and North and South America (http://bigsdb.pasteur.fr/klebsiella/klebsiella.html) and is the dominant clone carrying *bla*_KPC_ in China (7).

The *wzi* allele, which encodes the Wzi outer membrane protein of* cps* cluster, and the virulence factors of strain WCHKP649 were identified using the tools at http://bigsdb.pasteur.fr/klebsiella/klebsiella.html. Strain WCHKP649 had a *wzi* allele of type 64, which corresponds to the K14 and K64 serotypes. *K. pneumoniae* of K64 and ST11 has been found to be dominant clone among colistin-resistant isolates in Taiwan (8). Nonetheless, strain WCHKP649 was susceptible to colistin (MIC, 1 μg/ml). Strain WCHKP649 had a number of virulence factors, including yersiniabactin-encoding genes (*fyuA*, *irp1*, *irp2,* and the *ybt* system [*ybtAEPQTUX* genes]) (9) and the *mrk* system (*mrkABCDFHIJ* genes), encoding type 3 fimbriae for adherence (10).

ResFinder from the Center for Genomic Epidemiology (CGE; http://genomicepidemiology.org/) was employed to predict antimicrobial resistance genes. Strain WCHKP649 had the carbapenemase gene *bla*_KPC-2_ and the 16S rRNA methylase gene *rmtB*. The two genes are able to explain the high-level carbapenem- and amikacin-resistant phenotype. Other antimicrobial resistance genes in strain WCHKP649 included *bla*_CTX-M-65_ (mediating resistance to cephalosporins and penicillins), *bla*_TEM-1b_ and *bla*_SHV-11_ (resistance to penicillins), *catA2* (resistance to phenicol), *fosA* (resistance to fosfomycin), and *oqxA* and* oqxB* (resistance to quinolones). Plasmid replicons in strain WCHKP649 were predicted using PlasmidFinder at CGE. Strain WCHKP649 had four plasmid replicons, including IncFII, IncR, and two ColRNAI. *bla*_KPC-2_ is located on a 3,600-bp contig, which contains no plasmid replicon. Although *bla*_KPC-2_ has been found on IncFII plasmid in our local settings (11), the exact location of *bla*_KPC-2_ in strain WCHKP649 remains undetermined and warrants further investigation.

### Accession number(s).

This whole-genome shotgun project has been deposited at DDBJ/EMBL/GenBank under the accession no. MPOC00000000. The version described in this paper is the first version, MPOC01000000.
